# Feature Selection and Classification of Clinical Datasets Using Bioinspired Algorithms and Super Learner

**DOI:** 10.1155/2021/6662420

**Published:** 2021-05-17

**Authors:** S. Murugesan, R. S. Bhuvaneswaran, H. Khanna Nehemiah, S. Keerthana Sankari, Y. Nancy Jane

**Affiliations:** ^1^Ramanujan Computing Centre, Anna University, Chennai 600025, India; ^2^Department of Computer Science and Engineering, Anna University, Chennai 600025, India; ^3^Department of Computer Technology, Anna University, Chennai 600025, India

## Abstract

A computer-aided diagnosis (CAD) system that employs a super learner to diagnose the presence or absence of a disease has been developed. Each clinical dataset is preprocessed and split into training set (60%) and testing set (40%). A wrapper approach that uses three bioinspired algorithms, namely, cat swarm optimization (CSO), krill herd (KH) ,and bacterial foraging optimization (BFO) with the classification accuracy of support vector machine (SVM) as the fitness function has been used for feature selection. The selected features of each bioinspired algorithm are stored in three separate databases. The features selected by each bioinspired algorithm are used to train three back propagation neural networks (BPNN) independently using the conjugate gradient algorithm (CGA). Classifier testing is performed by using the testing set on each trained classifier, and the diagnostic results obtained are used to evaluate the performance of each classifier. The classification results obtained for each instance of the testing set of the three classifiers and the class label associated with each instance of the testing set will be the candidate instances for training and testing the super learner. The training set comprises of 80% of the instances, and the testing set comprises of 20% of the instances. Experimentation has been carried out using seven clinical datasets from the University of California Irvine (UCI) machine learning repository. The super learner has achieved a classification accuracy of 96.83% for Wisconsin diagnostic breast cancer dataset (WDBC), 86.36% for Statlog heart disease dataset (SHD), 94.74% for hepatocellular carcinoma dataset (HCC), 90.48% for hepatitis dataset (HD), 81.82% for vertebral column dataset (VCD), 84% for Cleveland heart disease dataset (CHD), and 70% for Indian liver patient dataset (ILP).

## 1. Introduction

Data related to symptoms observed on a patient at a point of time are stored in electronic health records (EHRs). Interesting patterns can be extracted from the data that are stored in EHRs, and the extracted patterns can be represented as knowledge, and this knowledge can assist the physicians to diagnose the presence or absence of a disease. Data mining tasks, namely, association rule mining, classification, and clustering are used to mine valuable patterns from the data stored in EHRs. Clinical decision support systems (CDSSs) that assist the physicians to diagnose the presence or absence of a disease can be developed from data stored in EHRs using bioinspired algorithms and data mining techniques. Although several algorithms have been proposed by researchers for association rule mining, classification, and clustering, no algorithm can be deliberated to be the “universal best.” Quality of data and data distribution are the two key factors that determine the effectiveness of a data mining task. The performance of a data mining task depends on how effective data preprocessing has been done. Classification plays a major role in the development of CDSSs. Classification is a two-step process, first, building the classifier and second, model usage. Building the classifier is the process of training the classifier with a supervised learning algorithm. Model usage is the process of estimating the accuracy of the classifier using testing instances commonly referred to as testing set. Overfitting and underfitting are two major problems associated with building the classifier.

Clinical dataset (*s*) (*C*_*s*_)  used for classifier construction is split into a training set (*T*_*s*_) and a testing set (*T*_*t*_). Researchers have proposed different methods to identify the *T*_*s*_ and *T*_*t*_. One common method is to split 80% of the dataset into *T*_*s*_ and 20% of the dataset into *T*_*t*_. For clinical decision-making, a balanced dataset is essential for building a prediction model. Clinical datasets are normally not balanced, and classification methods perform poorly on minority class samples when the dataset is tremendously imbalanced. For example, consider a *C*_*s*_ with *n* instances, each instance associated with a class label *c*_1_ or *c*_2_. Among the *n* instances that 75% of the instances in *C*_*s*_ are associated with class label *c*_1_, and 25% of the instances in *C*_*s*_ are associated with class label *c*_2_, it is evident that the class labels in *C*_*s*_ are not equally represented and therefore, the *C*_*s*_  is imbalanced. In this context, *c*_1_  is the majority class, and *c*_2_  is the minority class, and hence, constructing a classifier with class-imbalanced data will lead to bias in favor of the majority class. One method to handle class imbalance in a *C*_*s*_ is to generate additional instances from the minority class. The Synthetic Minority Oversampling Technique (SMOTE) [1] is one of the prevailing methods used to generate additional training and testing instances.

A training instance can be defined as a tuple *t*_*i*_(*f*_1_, *f*_2_, ⋯*f*_*m*_), where *t*_*i*_ represents a training instance, and (*f*_1_, *f*_2_, ⋯*f*_*j*_) represents the features corresponding to a training instance. The subscript *i* in *t*_*i*_ can range from 1 to *n*, where *n* is the number of instances. The subscript *j* in *f*_*j*_ can range from 1 to *m*, where *m* is the number of features. Using irrelevant features to train a classifier will affect its performance. Selecting the optimal features from the *C*_*s*_ and then training the classifier will enhance the accuracy of the classifier. Feature selection methods can be supervised, unsupervised, and semisupervised depending upon whether the training set is labeled or not. Commonly used supervised feature selection methods are filter and wrapper methods. The filter method considers the dependency of each feature to the class label and is independent of any classification algorithm. Measures, namely, information gain [2], gain ratio [3], Gini index [4], Laplacian score [5], and cosine similarity [6] can be used to rank the features. Other measures to rank the features can also be used in filter method. The wrapper method considers the classification accuracy of a learning algorithm to select the relevant features. Researchers are using a confluence of disciplines to develop computer-aided diagnostic (CAD) systems to assist physicians.

Knowledge mining using rough sets for feature selection and backpropagation neural network (BPNN) for classifying clinical datasets has been proposed in [7]. A CDSS to diagnose Urticaria using Bayes classification is proposed in [8]. CDSSs to diagnose lung disorders are proposed in [9–14]. A CDSS to diagnose the severity of gait disturbances using a *Q*-backpropogated time delay neural network on patients affected by Parkinson's disease is proposed in [15]. A statistical tolerance rough set induced decision tree classifier to classify multivariate time series clinical data is proposed in [16]. A CDSS to diagnose gestational diabetes mellitus using the fuzzy logic and radial basis function neural network is proposed in [17]. Use of fuzzy sets and extreme learning machine to classify clinical datasets is proposed in [18]. Wind-driven swarm optimization, a metaheuristic method to classify clinical datasets, is proposed in [19]. A computer-aided diagnostic system that uses a neural network classifier trained using differential evolution, particle swarm optimization, and gradient descent backpropagation algorithms is proposed in [20]. A radial basis function neural network to classify clinical datasets using *k*-means clustering algorithm and quantum-behaved particle swarm optimization is proposed in [21]. Classifying clinical unevenly spaced time series data by imputing missing values has been proposed in [22]. A framework to classify unevenly spaced time series clinical data using improved double exponential smoothing, rough sets, neural network, and fuzzy logic is proposed in [23].

An outline of nature-inspired algorithms for optimization is presented in [24]. The cooperative intellectual actions of insects or animal groups in nature, for example, colonies of ants, schools of fish, flock of birds, swarms of bees, and termites, have fascinated the thoughtfulness of researchers. Entomologists have studied the collective actions of insects or animals to model biological swarms, and engineers have applied these models as a framework to solve complex real-world problems.

In this work, a CAD system that employs a super learner to diagnose the presence or absence of a disease has been proposed. The bioinspired algorithms used in this work are cat swarm optimization (CSO), krill herd (KH), and bacterial foraging optimization (BFO). The classifiers used in this work are support vector machine (SVM) and BPNN trained using the conjugate gradient algorithm.

The rest of the paper is organized as follows: the abbreviation used in the manuscript is presented in Section 2. An outline of the related work is presented in Section 3. An outline of the datasets used is presented in Section 4. The framework of the proposed classifier is presented in Section 5. The results and discussions are presented in Section 6. Finally, conclusion and scope for future work are presented in section 7.

## 2. Abbreviations Used

Table 1 presents the abbreviation used in the rest of the manuscript in alphabetic order.

## 3. Literature Survey

Leema et al. [25] in their work have experimented the significance of fixing the appropriate values of parameters to train artificial neural networks using the backpropagation algorithm. The parameters are initial weight selection, bias, activation function used, number of hidden layers, number of neurons per hidden layer, number of training epochs, minimum error, and momentum term. Twelve backpropagation learning algorithms have been used in this study. Experimentation has been carried out using three clinical datasets from the UCI ML repository, namely, PID, hepatitis, and WBC datasets.

Elgin et al. [26] in their work have proposed a clinical-decision making system to diagnose allergic rhinitis. A wrapper approach that uses GA and the accuracy of ELM classifier as the fitness function has been used for feature selection. The selected features have been trained using ELM classifier. Intradermal skin test dataset of 872 patients collected from Good Samaritan Lab Services and Allergy Testing Centre, Chennai, has been used in this work, and an accuracy of 97.7% has been achieved.

Sreejith et al. [27] in their work have proposed a framework for classifying clinical datasets which uses an embedded approach for feature selection and a DISON for classification. The feature selection is performed by computing the feature importance of every attribute using an extremely randomized tree classifier. Classification is performed using DISON which is a feed forward neural network whose weights and bias are optimized in two stages first, by using a strawberry optimization algorithm and then by using a gradient descent BP algorithm. Vertebral column, PID, CHD, and SHD datasets from the UCI ML repository have been used for experimentation. The framework has achieved an accuracy of 87.17% for vertebral column, 90.92% for PID, 93.67% for CHD, and 94.5% for SHD.

Sreejith et al. [28] in their work have proposed a framework for CDSS which addresses the data imbalance problems associated with clinical dataset. The datasets are rebalanced using SMOTE enhanced using Orchard's algorithm. The feature selection is performed using a wrapper approach where CMVO is used to select the feature subsets, and RF classifier is used to evaluate the goodness of the features. The arithmetic mean of MCC and *F*-score computed using the RF classifier is used as the fitness function. Finally, an RF classifier, comprising of 100 decision trees which uses information gain ratio as the split criteria, is used for classifying the clinical data. Three clinical datasets from the UCI ML repository, namely, ILP, TS, and PID datasets, have been used for experimentation. The proposed framework achieved 0.65 MCC, 0.84 *F*-score, and 82.46% accuracy for ILP; 0.74 MCC, 0.87 *F*-score, and 86.88% accuracy for TS; and 0.78 MCC, 0.89 *F*-score, and 89.04% accuracy for PID datasets.

Isaac et al. [29] in their work have proposed a CAD system to diagnose pulmonary emphysema from chest CT slices. Spatial intuitionistic fuzzy *C*-means clustering algorithm has been used to segment the lung parenchyma and extracting the RoIs. From the RoIs, shape, texture, and run-length features have been extracted, and feature selection has been performed using a wrapper approach using four bioinspired algorithms with the classification accuracy of SVM as the fitness function. The bioinspired algorithms used are MFO, FFO, ABCO, and ACO. Tenfold crossvalidation technique has been used, and each feature set has been trained using an ELM classifier. Two independent datasets, one dataset consisting of CT slices collected from hospitals and the second dataset consisting of CT slices from a benchmark repository, have been used for classification. A maximum classification accuracy of 89.19% for MFO, 91.89% for FFO, 83.78% for ABCO, 86.49% for ACO, and 75.68% without feature selection have been achieved.

Elgin et al. [30] in their work have performed feature selection and instance selection using a wrapper approach that employs cooperative coevolution with the classification accuracy of the random forest classifier as the fitness function. The optimal feature set is used to train a random forest classifier. Seven datasets, namely, WDBC, HD, PID, CHD, SHD, VCD, and HCC from the UCI ML repository have been used for experimentation. An accuracy of 97.1%, 82.3%, 81.01%, 93.4%, 96.8%, 91.4%, and 72.2% for datasets WDBC, HD, PID, CHD, SHD, VCD, and HCC datasets have been achieved, respectively.

Anter et al. [31] in their work have developed CFCSA by integrating chaos theory and the FCM method to find the optimal feature subset. Ten clinical datasets from the UCI ML repository have been used for experimentation. The features of each clinical dataset have been normalized, and then random chaotic motion has been incorporated into CFCSA in the form of chaotic maps. The objective function of the FCM has been used as the fitness function, in which the crow with the best fitness has been considered the best solution. Comparison has been done with chaotic ant lion optimization, binary ant lion optimization, and the binary crow search algorithm, and it has been inferred that CFCSA outperforms these algorithms in all the datasets used for experimentation.

Elgin et al. [32] in their work have proposed a correlation-based ensemble feature selection using a wrapper approach that employs three bioinspired algorithms using differential evolution, lion optimization, and glowworm swarm optimization with the accuracy of the AdaboostSVM classifier as the fitness function. Tenfold crossvalidation technique has been used, and the optimal features selected have been used to train a gradient descent BP neural network with variable learning rates. Two clinical datasets from the UCI ML repository, namely, hepatitis and WDBC have been used for experimentation. An accuracy of 93.902% for hepatitis and 98.734% for WDBC datasets have been achieved.

Sweetlin et al. [33] in their work have proposed a CAD system to diagnose pulmonary tuberculosis from chest CT slices. The region growing algorithm has been used for segmenting the lung fields followed by edge reconstruction. The manifestations of pulmonary tuberculosis, namely, cavities, consolidations, and nodules have been considered to be RoIs. After extracting the RoIs, and from the RoI, texture features, run-length features and shape features have been extracted, and feature selection has been performed using a wrapper approach that employs the BCS algorithm with the accuracy of one-against-all multiclass SVM classifier as the fitness function. The Cuckoo search algorithm has been implemented in two ways, first, by using entropy measure and second, without using entropy measure. Using the selected feature training is performed using one-against-all multiclass SVM classifier. An accuracy of 85.54% for BCS algorithm with entropy measure and 84.65% accuracy for BCS algorithm without entropy measure have been achieved.

Sweetlin et al. [34] in their work have proposed a CAD system to diagnose pulmonary hamartoma nodules from chest CT slices. Otsu's thresholding method has been used to segment lung parenchyma from the CT slices. Nodules are considered to be the RoIs and from the RoIs, texture features, shape features and run-length features have been extracted. Feature selection has been performed using filter evaluation measures, namely, CSM and RDM with the ACO algorithm. The features selected by ACO-CSM and ACO-RDM have been used to train three classifiers, namely, SVM, NB, and J48 decision tree classifiers. Maximum classification accuracy of 94.36% for SVM classifier trained with 38 features selected using ACO-RDM has been achieved.

Sweetlin et al. [35] in their work have proposed a CAD system to diagnose pulmonary bronchitis from CT slices of the lung. Optimal thresholding has been used to segment the left and right lung fields from the lung CT slices. The RoIs are identified, and from the RoIs, texture and shape features have been extracted. Feature selection has been performed using a hybrid ACO algorithm combined with tandem run recruitment based on cosine similarity, and the accuracy of the SVM classifier has been used as the fitness function. The selected features have been used to train a SVM classifier. An accuracy of 81.66% for ACO with tandem run strategy, 78.10% for ACO without tandem run strategy, and 75.14% without feature selection has been achieved.

Raj et al. [36] in their work have proposed DGA for feature selection to develop a CAD system to diagnose lung disorders from chest CT slices. The entire dataset has been split into two sets one set containing 90% of the entire dataset and the other set containing 10% of the entire dataset. Out of the 90%, 50% has been used as training set and the other 50% as validation set for evaluating the objective function. The set containing 10% of the entire dataset has been used as testing set. The objective function has been defined as the sum of the squared deviation of each data in the training set of each class from each data in the validation set of the corresponding class. GA has been used for feature selection by minimizing the proposed objective function, resulting in the proposed DGA. The GA has been iterated over several generations to obtain individuals that are best fit with respect to the objective function. Classification has been performed using *k*-NN classifier to classify the RoIs into one of four classes, namely, bronchiectasis, tuberculosis, pneumonia, and normal. An average accuracy of 88.16% with feature selection and an average accuracy of 86.46% without feature selection have been achieved.

Zawbaa et al. [37] in their work have performed feature selection using a wrapper approach that uses the MFO algorithm with the accuracy of *k*-NN classifier as the fitness function. Eighteen datasets from the UCI ML repository have been used for experimentation among which four are clinical datasets. Comparison has been done with PSO and GA, and it has been inferred that MFO outperforms in fourteen datasets among which three are clinical datasets.

Shu-Chuan et al. [38] in their work have presented an algorithm called CSO by modeling the natural behavior of cats. The CSO algorithm considered two biological characteristics of cats, namely, seeking mode and tracking mode. Cats spend utmost of the time when they are awake on resting. Nevertheless, during their rests, their perception is really high, and they are well aware of what is happening around them. Cats continuously observe their environment wisely and consciously and when they perceive a prey, they advance towards it rapidly. Although resting, they move their position cautiously and slowly, occasionally even stay in the original position. Seeking mode has been used to represent this behavior into the CSO, and the tracing mode has been used to represent the behavior of cats advancing towards a prey into the CSO. The performance of CSO has been evaluated by applying CSO, standard PSO, and PSO with weighting factor into six benchmark functions. The results obtained reveal that the proposed CSO performs better compared to PSO and PSO with weighting factor.

Gandomi et al. [39] in their work have proposed a swarm intelligence algorithm named KH algorithm to solve optimization tasks and is centered on the imitation of the herding behavior of krill swarms with respect to precise biological and environmental processes. The fitness function of each krill individual has been defined as the least distance of each individual krill from food and from the highest density of the herd. Three vital actions considered to define the time-dependent position of an individual krill are, one, movement induced by other krill individuals, two, foraging activity, and three, random diffusion. The KH algorithm is tested using twenty benchmark functions and compared with eight algorithms. Experimentation results indicate that the KH algorithm can outperform these familiar algorithms.

Chen et al. [40] have proposed a cooperative bacterial foraging optimization algorithm (CBFO). Two cooperative methods are used to solve complex optimization problems in the original BFO [41] and achieved significant improvement. The serial heterogeneous cooperation on the implicit space decomposition level and the hybrid space decomposition level are the two methods used to improve the original BFO. The authors have compared the performance of two CBFO variants with the original BFO, PSO, and GA on four commonly used benchmark functions. The experimental results indicated that the CBFO achieved a better performance over the original BFO, PSO, and GA.

Chen et al. [42] have proposed an adaptive bacterial foraging optimization (ABFO) for optimizing functions. The adaptive foraging approaches are used to increase the performance of the original BFO. It is achieved by enabling the original BFO to adjust the run-length unit parameter dynamically during the time of algorithm implementation. The experimental results are compared with the original BFO, PSO, and GA using 4 benchmark functions. The proposed ABFO indicates the better performance over the original BFO and competitive with the PSO and GA.

From the literature, it is evident that classifier training using relevant features enhances the accuracy of the classifier. It can also be inferred that wrapper-based feature selection that employs bioinspired algorithms performs better in numerous cases compared to traditional feature selection methods.

## 4. Outline of the Datasets Used

Seven clinical datasets from the UCI ML repository, namely, WDBC, SHD, HCC, HD, VCD, CHD, and ILP have been used for binary classification. An outline of each dataset used is presented in Table 2.

## 5. System Framework

**Figure 1:**
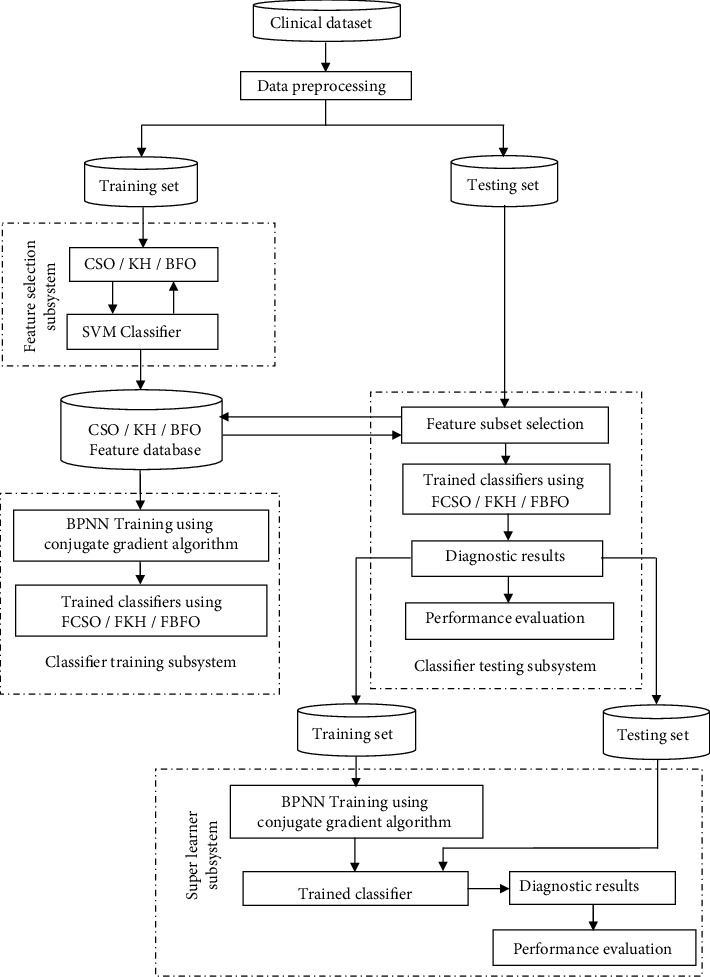
System framework.

The framework for feature selection and classification of clinical datasets using bioinspired algorithms and super learner is presented in Figure 1. The major building blocks of the framework are data preprocessing, feature selection, classifier training, classifier testing, and dataset construction for super learner, super learner training, and testing. Each building block is outlined below.

### 5.1. Preprocessing

Each (*C*_*s*_) has been subjected to preprocessing prior to feature selection to enhance the quality of data. Mean imputation has been used to handle missing values, and SMOTE is used to handle the class imbalance problem in each *C*_*s*_ by generating additional instances from the minority class.

Normalization has been used to scale the value of a feature so that the value will fall in a specified range and is predominantly useful for constructing a classifier involving a neural network. Training a classifier using normalized data will speedup learning. In this work, the range is 0 to 1, and min-max normalization is being used. When an attribute “*A*” in a clinical dataset *C*_*s*_ is subject to min-max normalization, the minimum value (min_*A*_) and maximum value (max_*A*_) in the value set of “*A*” are first identified, and normalization is performed using the formula presented in equation (1).

(1) $$\begin{array}{c} a^{\prime }=\frac{a-\min_{A}}{\max_{A}-\min_{A}}\;\left({new}_{\max_{A}}-{new}_{\min_{A}}\right)+{new}_{\min_{A}}. \end{array}$$

If the formula “*a*′” is the normalized value of an attribute “*a*,” when *a* is drawn from the value set of “*A*.” Since min-max normalization is being used to normalize the values in the range 0 to 1, the value of new_max_*A*__ is 1 and new_min_*A*__ is 0.

The number of instances in each *C*_*s*_ used for constructing and testing the classifier prior to generating additional samples using SMOTE, the number of instances in each *C*_*s*_ after generating additional samples using SMOTE, the number of instances in the training set (*T*_*s*_), and the number of instances in the testing set (*T*_*t*_) is presented in Table 3. After preprocessing, each *C*_*s*_ is split into training set (60%) and testing set (40%).

### 5.2. Feature Selection

Feature selection is performed on each *T*_*s*_ used for experimentation to select the optimal features for training the classifier. Selecting the optimal features from the *T*_*s*_ will improve the classification accuracy. A wrapper approach that uses three bioinspired algorithms, namely, CSO, KH, and BFO with the accuracy of the SVM classifier is used to perform feature selection. An outline of CSO, KH, and BFO used for feature selection is presented below.

#### 5.2.1. Outline of the CSO Algorithm for Feature Selection

CSO is inspired and modeled based on two main postures of cats, namely, resting and tracing. Mimicking the resting behavior of a cat is named as seeking mode, and mimicking the tracing behavior of a cat is named as tracing mode. The seeking mode relates to a local search process, whereas the tracing mode relates to a global search process. The vital parameters that play an important role in CSO are outlined in Table 4. Tracing mode relates to cat's movement while chasing a prey, for example, chasing a rat.

The steps to select the optimal feature subset using CSO is outlined below (Algorithm 1):

#### 5.2.2. Outline of the KH Algorithm for Feature Selection

The KH algorithm is centered on the imitation of the herding behavior of krill swarms with respect to precise biological and environmental processes. Krill density is reduced by predators, namely, seals, penguins, or seabirds. The herding of the krill individuals includes, one, increasing the krill density and two, reaching the food. The fitness function of each krill individual has been defined as the least distance of each individual krill from food and from the highest density of the herd.

Three vital actions considered to define the time-dependent position of an individual krill are one, movement induced by other krill individuals, two, foraging activity, and three, random diffusion.

Krill individuals attempt to maintain a high density and hence move due to their mutual effect. Local swarm density, target swarm density, and repulsive swarm density are used to estimate the direction of motion. Food location and prior experience about the food location are the two parameters used to estimate the foraging motion. Random diffusion is used for the exploration of the search space. In the KH algorithm, the population diversity is improved by means of the diffusion function, which is integrated into the krill individuals. Random diffusion is the net movement of each krill individual from high-density to low-density regions.

The motion velocity of krill particle applies the Lagrangian model [43] as shown in Equation (5).

(5) $$\begin{array}{c} \frac{{dx}_{i}}{dt}=N_{i}+F_{i}+{\text{RD}}_{i}. \end{array}$$

In the above formula, *dx*_*i*_/*dt* is the motion velocity of krill particle *i*, *N*_*i*_ is the induced motion, *F*_*i*_  is the foraging motion, and RD_*i*_ is the random diffusion of the *i*^*th*^ krill individual. The vital parameters that play an important role in the KH algorithm are outlined in Table 5.

The steps to select the optimal feature subset using KH is outlined below (Algorithm 2):

#### 5.2.3. Outline of the BFO Algorithm for Feature Selection

The bacterial foraging optimization (BFO) algorithm imitates the pattern exhibited during the foraging process of Escherichia coli bacteria, that includes chemotaxis, swarming, reproduction, and elimination-dispersal operations [41]. The basic idea behind the foraging strategy of E. coli bacteria is to obtain the maximum nutrition in a unit time. The chemotaxis strategy involves the searching of nutrition by taking small movements such as tumbling, moving, and swimming, using its locomotory organ called flagella. The swarming strategy deals with the communication between bacteria. When the bacteria discover high amount of nutrients, they will release chemical substances to attract other bacteria. If they are in danger, they will tend to prevent other bacteria. The reproduction process involves splitting of healthier bacterium into two bacteria, and the low healthy bacteria are set to die. Finally, the elimination-dispersal strategy involves replacing the low health bacterium by randomly generated new ones. The vital parameters that play an important role in the BFO algorithm are outlined in Table 6.

The steps involved in finding the optimal feature subset using the BFO algorithm is outlined below:

### 5.3. Classifier Training

**Figure 2:**
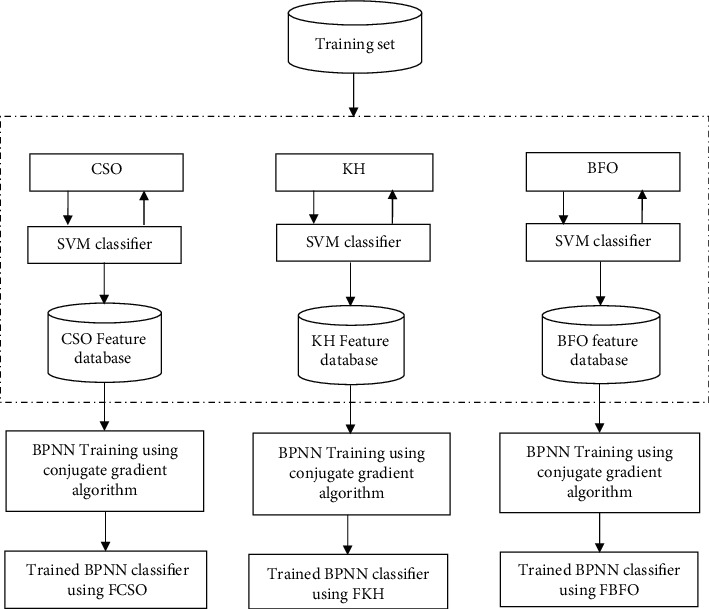
Classification using BPNN.

Each *C*_*s*_  is preprocessed and split into training set (*T*_*s*_ − 60%) and testing set (*T*_*t*_− 40%). A wrapper approach that uses three bioinspired algorithms CSO, KH, and BFO with the classification accuracy of SVM as the fitness function has been used for feature selection. The features selected by each bioinspired algorithm are used to train three BPNNs independently using CGA. The number of hidden layers for each BPNN is 1, and the activation function used in the hidden layer is sigmoid. The learning rate is 1*e*–07, and the maximum number of iterations is 100. Since the classification is binary, each BPNN has only one output node, and the activation function used in the output layer is sigmoid.  Figure 2 elaborates the process of training BPNN classifiers.

The number of training instances for FCSO, FKH, and FBFO classifiers is presented in Table 3. Though majority of the features selected by each bioinspired algorithm overlap, it has been inferred that the number of features selected by each algorithm is not the same. The parameter settings for each classifier is presented in Table 7.

The steps to train the BPNN classifier using three BPNN classifier and trained using CSO, KH, and BFO algorithms are outlined below:

### 5.4. Classifier Testing and Dataset Construction for Super Learner

**Figure 3:**
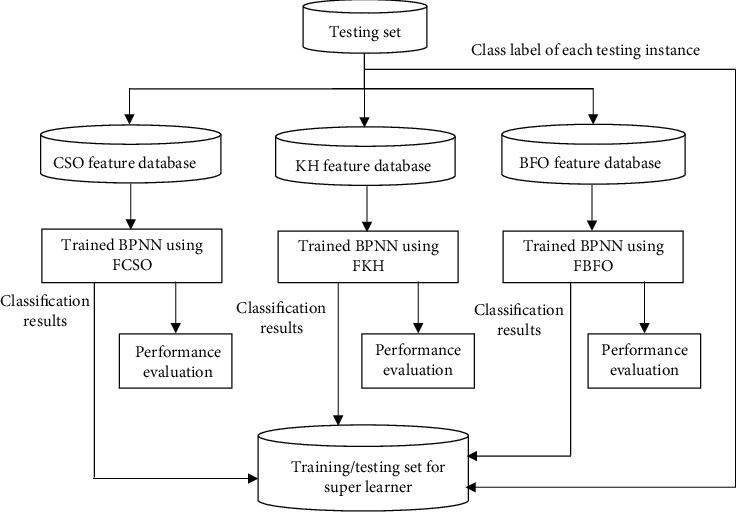
Classifier testing.

After training the classifier with 60% of the preprocessed *C*_*s*_ (*T*_*s*_), classifier testing is performed using the remaining 40% of the of the preprocessed *C*_*s*_ (*T*_*t*_).  Figure 3 elaborates the process of testing the three classifiers and also throws light on the process of training the super learner.

Feature selection is performed on the testing set by querying the FCSO, FKH, and FBFO databases. The instances of the testing set containing the features selected by the CSO are used to test the FCSO classifier; similarly, the instances of the testing set containing the features selected by the KH and BFO are used to test the FKH and FBFO classifier. The performance of the FCSO, FKH, and FBFO classifiers are evaluated using the results obtained from the testing set.

The classification result of each instance of the testing set for FCSO, FKH, and FBFO classifiers and the class label corresponding to each instance of the testing set will be the candidate instances for training and testing the super learner.

### 5.5. Super Learner Training and Testing

**Figure 4:**
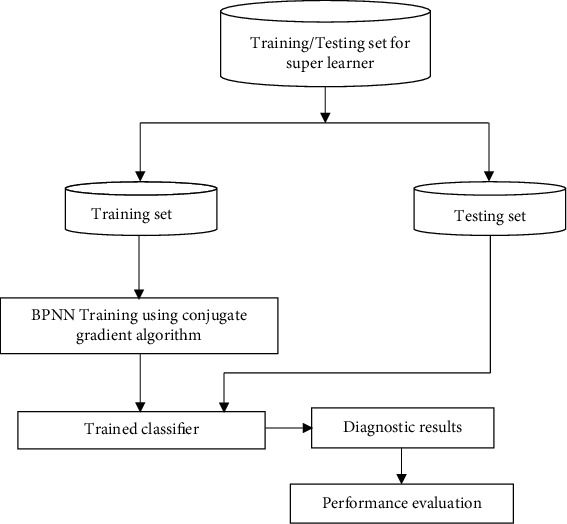
Super learner training and testing.

As outlined in Section 5.4, the classification result pertaining to each instance of the testing set for FCSO, FKH, and FBFO classifiers and the class label corresponding to each instance of the testing set will be the candidate instances for training and testing the super learner.  Figure 4 elaborates the process of training and testing of the super learner. The training set comprises of 80% of the instances, and the testing set comprises of 20% of the instances. The number of training and testing instances for the super learner is presented in Table 3.

Super learner is a type of ensemble classifier [44]. In this work, a BPNN classifier trained using CGA is used as the super learner. The parameter settings for the super learner are presented in Table 8.

The super learner is trained using the steps presented in Section 5.3 for training the BPNN classifier using CGA, and the performance of the super learner is evaluated using the testing set.

## 6. Results and Discussions

Seven clinical datasets from the UCI ML repository, namely, WDBC, SHD, HCC, HD, VCD, CHD, and ILP have been used for experimentation. The performance of the FCSO, FKH, and FBFO classifiers and super learner is evaluated in terms of accuracy, sensitivity, specificity, precision, and *F*-score, which are calculated based on true positive (TP), true negative (TN), false positive (FP), and false negative (FN) using Equations (22), (23), (24), (25), and (26).

(22) $$\begin{array}{c} \text{Accuracy}=\frac{\text{TP}+\text{TN}}{\text{TP}+\text{TN}+\text{FP}+\text{FN}}. \end{array}$$

In the above formula, TP is the number of positive instances predicted as positive by the classifier, TN is the number of negative instances predicted as negative by the classifier, FP is the number of negative instances predicted as positive by the classifier, and FN is the number of positive instances predicted as negative by the classifier.

(23) $$\begin{array}{c} \text{Sensitivity}=\frac{\text{TP}}{\text{TP}+\text{FN}}, \end{array}$$

(24) $$\begin{array}{c} \text{Specificity}=\frac{\text{TN}}{\text{TN}+\text{FP}}, \end{array}$$

(25) $$\begin{array}{c} \text{Precision}=\frac{\text{TP}}{\text{TP}+\text{FP}}, \end{array}$$

(26) $$\begin{array}{c} \text{F}-\text{score}=\frac{2\text{TP}}{2\text{TP}+\left(\text{FP}+\text{FN}\right)}. \end{array}$$

Accuracy, sensitivity, specificity, precision, and *F*−score obtained using FCSO, FKH, and FBFO classifiers and super learner for the datasets WDBC, SHD, HCC, HD, VCD, CHD, and ILP are presented in Tables 9–15.

The super learner has achieved a classification accuracy of 96.83% for WDBC, 86.36% for SHD, 94.74% for HCC, 90.48% for HD, 81.82% for VCD, 84.0% for CHD, and 70.0% for ILP. The classification accuracy of the proposed work has been compared with the performance of the existing work on clinical datasets and the comparison results summarized in Table 16.

## 7. Conclusion and Scope for Future Work

A CAD system that employs a super learner to diagnose the presence or absence of a disease has been implemented in this work. Seven *C*_*s*_ from the UCI ML repository, namely, WDBC, SHD, HCC, HD, VCD, CHD, and ILP have been used for experimentation. Each *C*_*s*_ is preprocessed, and the preprocessed *C*_*s*_ is split into training and testing sets. A wrapper-based feature selection approach using three bioinspired algorithms, namely, CSO, KH, and BFO, with the accuracy of SVM classifier has been used to select the optimal feature subsets. The selected feature subsets are used to train three BPNN classifiers using CGA, and the performance of the trained classifiers is evaluated. The classification results obtained for each instance of the testing set of the three classifiers and the class label associated with each instance of the testing set will be the candidate instances for training and testing the super learner. The super learner achieved a classification accuracy of 96.83% for WDBC, 86.36% for SHD, 94.74% for HCC, 90.48% for HD, 81.82% for VCD, 84.0% for CHD, and 70.0% for ILP.

CAD systems to diagnose disorders in the human body from different imaging modalities such as X-ray, computed tomography, magnetic resonance imaging, and positron emission tomography are gaining importance. This work can be extended by developing CAD systems to diagnose disorders from the medical images acquired through different imaging modalities. Features based on shape, texture, and run length can be extracted from the images, and the feature selection algorithms used in this work can be used to select the relevant features. The relevant features can be used to build classifier models to predict the presence or absence of disorders from the images.

## Figures and Tables

**Algorithm 1:**
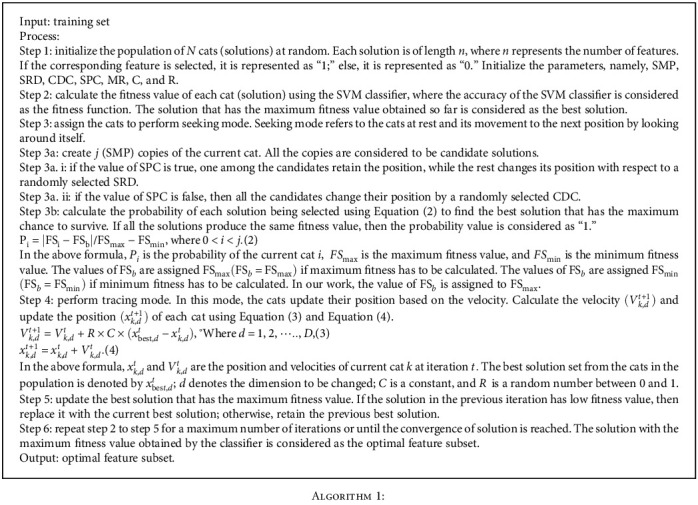


**Algorithm 2:**
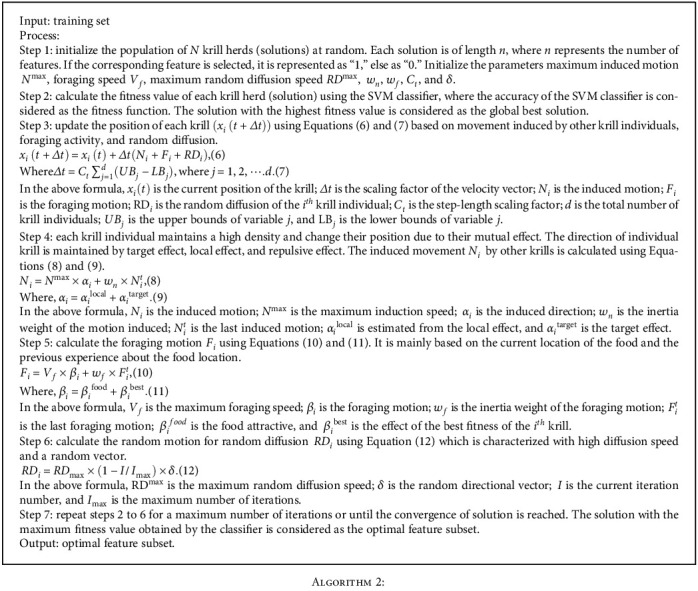


**Algorithm 3:**
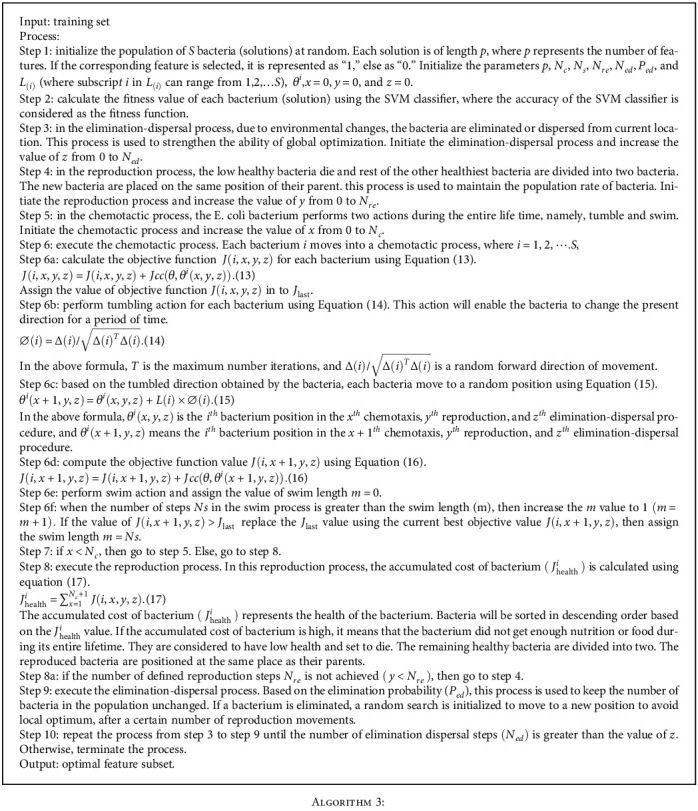


**Algorithm 4:**
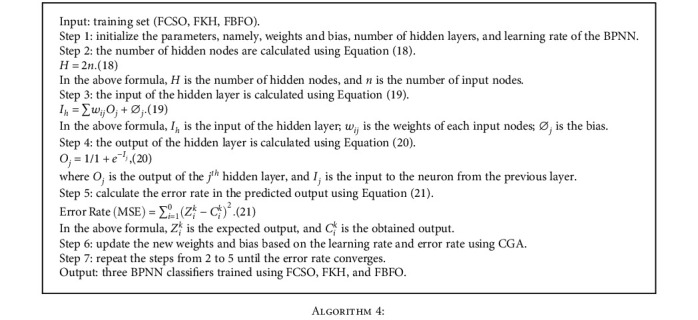


**Table 1:** Abbreviations used.

Abbreviation	Phrase
ABCO	Artificial bee colony optimization
ACO	Ant colony optimization
ANN	Artificial neural networks
BCS	Binary cuckoo search
BFA	Binary firefly algorithm
BFO	Bacterial foraging optimization
BP	Back propagation
BPNN	Back propagation neural network
CAD	Computer-aided diagnosis
CDC	Counts of dimension to change
CDSS	Clinical decision support system
CFCSA	Hybrid crow search optimization algorithm
CGA	Conjugate gradient algorithm
CHD	Cleveland heart disease
CMVO	Chaotic multiverse optimization
CSM	Cosine similarity measure
CSO	Cat swarm optimization
CT	Computed tomography
DE	Differential evolution
DGA	Distance-based genetic algorithm
DISON	Diverse intensified strawberry optimized neural network
DNN	Deep neural network
E.coli	Escherichia Coli Bacteria
ECSA	Enhanced crow search algorithm
ELM	Extreme learning machine
FBFO	Feature selected by bacterial foraging optimization
FCM	Fuzzy *C*-means
FCSO	Feature selected by cat swarm optimization
FFO	Firefly optimization
FKH	Feature selected by krill herd
GA	Genetic algorithm
GSO	Glowworm swarm optimization
HCC	Hepatocellular carcinoma
HD	Hepatitis
IBPSO	Improved binary particle swarm optimization
ILP	Indian liver patient
ISSA	Improved Salp swarm algorithm
KH	Krill herd
k-NN	*k*-nearest neighbors
LO	Lion optimization
LR	Logistic regression
MCC	Mathew's correlation coefficient
MFO	Moth-flame optimization
ML	Machine learning
MPNN	Multilayer perceptron neural network
MR	Mixed ratio
NB	Naive Bayes
PCC	Pearson correlation coefficient
PID	Pima Indian diabetes
PSO	Particle swarm optimization
RD	Random diffusion
RDM	Rough dependency measure
RF	Random forest
RoIs	Regions of interest
SHD	Statlog heart disease
SMOTE	Synthetic minority oversampling technique
SMP	Seeking memory pool
SPC	Self-position consideration
SRD	Seeking range of the selected dimension
SVC	Support vector classification
SVM	Support vector machine
TS	Thoracic surgery
UCI	University of California Irvine
VCD	Vertebral column dataset
WBC	Wisconsin breast cancer
WDBC	Wisconsin diagnostic breast cancer
WOA	Whale optimization algorithm

**Table 2:** Outline of the datasets used.

Dataset name	No. of instances	No. of features^∗^	No. of missing values	Class labels with no. of instances associated with each class label	Interpretation of class labels
WDBC	569	31	Nil	M (212)/B (357)	M-malignant, B-benign
SHD	270	13	Nil	2 (120)/1 (150)	2-present, 1-absent
HCC	165	49	826	0 (63)/1 (102)	0-dies, 1-lives
HD	155	18	167	1 (32)/2 (123)	1-die, 2-live
VCD	310	6	Nil	0 (210)/1 (100)	0-abnormal, 1-normal
CHD	303	13	Nil	1 (139)/2 (164)	1-presence, 2-absence
ILP	583	10	Nil	1 (416)/2 (167)	1-diseased, 2-nondiseased

^∗^ without class label.

**Table 3:** Outline of training and testing instances of each *C*_*s*_.

Instances	WDBC dataset	SHD dataset	HCC dataset	HD dataset	VCD dataset	CHD dataset	ILP dataset
Total number of instances before SMOTE	569	270	165	155	310	303	583
Total number of instances after SMOTE	780	270	228	251	410	303	750
Number of training instances for FCSO/FKH/FBFO classifiers	468	162	137	151	246	182	450
	60% of the total number of instances after SMOTE						
Number of testing instances for FCSO/FKH/FBFO classifiers	312	108	91	100	164	121	300
	40% of the total number of instances after SMOTE						
Number of training instances for super learner	250	86	73	80	131	97	240
	80% of the total testing instances^∗^ for FCSO/FKH/FBFO classifiers						
Number of testing instances for	62	22	18	20	33	24	60
Super learner	20% of the total testing instances^∗^ for FCSO/FKH/FBFO classifiers						

^∗^Each instance refers to the classification result pertaining to each instance of the testing set for FCSO, FKH, and FBFO classifiers and the class label corresponding to each instance of the testing set.

**Table 4:** Outline of the parameters used in CSO.

Parameter	Description
SMP	SMP is used to define the size of the seeking memory of each cat. Each cat selects possible neighborhood position from a set of solutions.
SRD	SRD is used to define the seeking range of the selected dimension.
CDC	CDC is a count of dimensions to be changed in seeking mode.
SPC	SPC indicates whether the cat is in the current position or not.
N	Number of cats
MR	Mixed ratio of cats
C	Constant value
D	Size of dimension
R	Random number in the range of [0,1]

**Table 5:** Outline of the parameters used in the KH algorithm.

Parameter	Definition	Value
*V*_*f*_	Maximum foraging speed	*V*_*f*_ = 0.02 m/s^−1^
RD^max^	Maximum random diffusion speed	RD^max^ ∈ (0.002 − 0.01 ) m/s^−1^
*N* ^max^	Maximum induction speed	*N* ^max^ = 0.01 m/s^−1^
*w*_*n*_	Inertia weight of the motion induced	*w*_*n*_ ∈ (0, 1)
*w*_*f*_	Inertia weight of the foraging motion	*w*_*f*_ ∈ (0, 1)
*C*_*t*_	Step-length scaling factor	Constant no.between [0, 2]
*δ*	Random directional vector	Random numbers [−1, 1]

**Table 6:** Outline of the parameters used in the BFO algorithm.

Parameter	Description
*p*	Number of features
*S*	Number of bacteria
*S*_*r*_	Number of bacteria in the reproduction steps
*N*_*re*_	No. of reproductive steps
*N*_*ed*_	No. of elimination-dispersal steps
*N*_*c*_	No. of chemotactic steps
*N*_*s*_	No. of swimming steps
*L*_(*i*)_	Bacteria step size length
*P*_*ed*_	Elimination probability
∅(*i*)	Direction of *i*^*th*^ bacteria
*x*	Index of the chemotactic process
*y*	Index of the reproduction process.
*z*	Index of the elimination-dispersal process
*θ* ^*i*^	The *i*^*th*^ bacterium position
*θ*	A bacterium on the optimization domain
*J*_last_	The highest objective function value
∆(*i*)	A random vector and its value lie between -1 and 1
*J*cc(*θ*, *θ*^*i*^(*x*, *y*, *z*))	Cell-to-cell attractant effect to nutrient concentration

**Table 7:** Parameter settings for BPNN.

BPNN parameter	Bioinspired algorithm	WDBC dataset	SHD dataset	HCC dataset	HD dataset	VCD dataset	CHD dataset	ILP dataset
Number of input nodes	CSO	15	9	20	16	3	6	5
	KH	17	10	39	10	3	10	8
	BFO	18	9	35	19	2	11	5
Number of hidden nodes	CSO	30	18	40	32	6	12	10
	KH	34	20	78	20	6	20	16
	BFO	36	18	70	38	4	22	10

**Table 8:** Parameter settings for super learner.

Name of the parameter	WDBC dataset	SHD dataset	HCC dataset	HD dataset	VCD dataset	CHD dataset	ILP dataset
Initial population size	250	86	73	80	131	97	240
Number of input nodes	3	3	3	3	3	3	3
Number of hidden nodes	6	6	6	6	6	6	6

**Table 9:** Performance of FCSO, FKH, and FBFO classifiers and super learner on WDBC dataset.

Feature selection algorithm	Size of feature subset	TN	FP	FN	TP	Accuracy	Sensitivity	Specificity	Precision	*F*-score
CSO	15	137	4	6	165	96.79	96.49	97.16	97.63	0.97
KH	17	139	2	5	166	97.76	97.08	98.58	98.81	0.98
BFO	18	139	2	8	163	96.79	95.32	98.58	98.79	0.97
Super learner	—	22	0	2	39	96.83	95.12	100.00	100.00	0.98

**Table 10:** Performance of FCSO, FKH, and FBFO classifiers and super learner on Statlog dataset.

Feature selection algorithm	Size of feature subset	TN	FP	FN	TP	Accuracy	Sensitivity	Specificity	Precision	*F*-score
CSO	9	53	8	9	38	84.26	80.85	86.89	82.61	0.82
KH	10	53	8	11	36	82.41	76.60	86.89	81.82	0.79
BFO	9	51	10	10	37	81.48	78.72	83.61	78.72	0.79
Super learner	—	10	1	2	9	86.36	81.82	90.91	90.00	0.86

**Table 11:** Performance of FCSO, FKH, and FBFO classifiers and super learner on HCC dataset.

Feature selection algorithm	Size of feature subset	TN	FP	FN	TP	Accuracy	Sensitivity	Specificity	Precision	*F*-score
CSO	20	43	9	9	31	80.43	77.50	82.69	77.50	0.78
KH	39	48	4	13	27	81.52	67.50	92.31	87.10	0.76
BFO	35	47	5	20	20	72.83	50.00	90.38	80.00	0.62
Super learner	—	10	1	0	8	94.74	100.00	90.91	88.89	0.94

**Table 12:** Performance of FCSO, FKH, and FBFO classifiers and super learner on hepatitis dataset.

Feature selection algorithm	Size of feature subset	TN	FP	FN	TP	Accuracy	Sensitivity	Specificity	Precision	*F*-score
CSO	16	47	2	10	42	88.12	80.77	95.92	95.45	0.88
KH	10	45	4	6	46	90.10	88.46	91.84	92.00	0.90
BFO	19	47	2	12	40	86.14	76.92	95.92	95.24	0.85
Super learner	—	8	1	1	11	90.48	91.67	88.89	91.67	0.92

**Table 13:** Performance of FCSO, FKH, and FBFO classifiers and super learner on vertebral column dataset.

Feature selection algorithm	Size of feature subset	TN	FP	FN	TP	Accuracy	Sensitivity	Specificity	Precision	*F*-score
CSO	3	74	10	17	63	83.54	78.75	88.10	86.30	0.82
KH	3	81	3	13	67	90.24	83.75	96.43	95.71	0.89
BFO	2	80	4	17	63	87.20	78.75	95.24	94.03	0.86
Super learner	—	19	2	4	8	81.82	66.67	90.48	80.00	0.73

**Table 14:** Performance of FCSO, FKH, and FBFO classifiers and super learner on Cleveland heart disease dataset.

Feature selection algorithm	Size of feature subset	TN	FP	FN	TP	Accuracy	Sensitivity	Specificity	Precision	*F*-score
CSO	6	60	8	12	42	83.61	77.78	88.24	84.00	0.81
KH	10	56	12	11	43	81.15	79.63	82.35	78.18	0.79
BFO	11	53	15	13	41	77.05	75.93	77.94	73.21	0.75
Super learner	—	13	2	2	8	84.00	80.00	86.67	80.00	0.80

**Table 15:** Performance of FCSO, FKH, and FBFO classifiers and super learner on Indian liver patient dataset.

Feature selection algorithm	Size of feature subset	TN	FP	FN	TP	Accuracy	Sensitivity	Specificity	Precision	*F*-score
CSO	5	103	62	33	102	68.33	75.56	62.42	62.20	0.68
KH	8	104	61	40	95	66.33	70.37	63.03	60.90	0.65
BFO	5	101	64	34	101	67.33	74.81	61.21	61.21	0.67
Super learner	—	26	15	3	16	70.00	84.21	63.41	51.61	0.64

**Table 16:** Comparison of the proposed work and existing work using clinical dataset.

Author/year	Method/reference	Accuracy %						
		WDBC	SHD	HCC	HD	VCD	CHD	ILP
Ayon et al. (2020)	DNN [45]	—	98.15	—	—	—	94.39	—
	SVM [45]	—	97.41	—	—	—	97.36	—
Bai Ji et al. (2020)	IBPSO with *k*-NN [46]	96.14	—	—	—	—	—	—
Elgin et al. (2020)	Cooperative coevolution and RF [30]	97.1	96.8	72.2	82.3	91.4	93.4	—
Magesh et al. (2020)	Cluster-based decision tree [47]	—	—	—	—	—	89.30	—
Rabbi et al. (2020)	PCC and AdaBoost [48]	—	—	—	—	—	—	92.19
Rajesh et al. (2020)	RF classifier [49]	—	—	80.64	—	—	—	—
Salima et al. (2020)	ECSA with *k*-NN [50]	95.76	82.96	—	—	—	—	—
Singh J et al. (2020)	Logistic regression [51]	—	—	—	—	—	—	74.36
Sreejith et al. (2020)	CMVO and RF [28]	—	—	—	—	—	—	82.46
Sreejith et al. (2020)	DISON and ERT[27]	—	94.5	—	—	87.17	93.67	—
Tougui et al. (2020)	ANN with Matlab [52]	—	—	—	—	—	85.86	—
Tubishat et al. (2020)	ISSA with k-NN [53]	—	88.1	—	—	89.0	—	—
Abdar et al. (2019)	Novel nested ensemble nu-SVC [54]	—	—	—	—	—	98.60	—
Anter et al. (2019)	CFCSA with chaotic maps [31]	98.6	—	—	68.0	—	88.0	68.4
Aouabed et al. (2019)	Nested ensemble nu-SVC, GA and multilevel balancing [55]	—	—	—	—	—	98.34	—
Elgin et al. (2019)	DE, LO and GSO with Adaboost SVM [32]	98.73	—	—	93.9	—	—	—
Książek et al. (2019)	SVM [56]	—	97.41	—	—	—	97.36	—
Sayed et al. (2019)	Novel chaotic crow search algorithm with *k*-NN [57]	90.28	78.84	—	83.7	—	—	71.68
Abdar et al. (2018)	MPNN and C5.0 [58]	—	—	—	—	—	—	94.12
Abdullah et al. (2018)	*k*-NN [59]	—	—	—	—	85.32	—	—
	RF [59]	—	—	—	—	79.57	—	—
Sawhney et al. (2018)	BFA and RF [60]	—	—	83.50	—	—	—	—
Abdar et al. (2017)	Boosted C5.0 [61]	—	—	—	—	—	—	93.75
	CHAID [61]	—	—	—	—	—	—	65.0
Zamani et al. (2016)	WOA with *k*-NN [62]	—	77.05	—	87.10	—	—	—
Abdar (2015)	SVM with rapid miner [63]	—	—	—	—	—	—	72.54
	C5.0 with IBM SPSS modeller [63]	—	—	—	—	—	—	87.91
Santos et al. (2015)	Neural networks and augmented set approach [64]	—	—	75.2	—	—	—	—
Chiu et al. (2013)	ANN and LR [65]	—	—	85.10	—	—	—	—
Mauricio et al. (2013)	ABCO with SVM [66]	—	84.81	—	87.10	—	83.17	—
Proposed	CSO, KH, BFO, and super learner	96.83	86.36	94.74	90.48	81.82	84.00	70.00

## Data Availability

The data supporting this study are from previously reported studies and datasets, which have been cited. The datasets used in this research work are available at UCI Machine Learning Repository.
